# An investigation of the effect of purslane (*Portulaca oleracea *L*.*) extract on body resistance toward thirst by examining urine and blood variables in laboratory mice

**Published:** 2020

**Authors:** Isa Jafari Foutami, Nafiseh Hosseini Yekta, Mozhgan Mehri

**Affiliations:** 1 *Department of Persian Medicine, Faculty of Medicine, AJA University of Medical Sciences, Tehran, Iran*

**Keywords:** Ethnobotany, Iranian traditional medicine, Portulaca oleracea L., Thirst

## Abstract

**Objective:**

*Portulaca oleracea* L. (PO) is abundantly found in Iran and is used in both nutritional and traditional medicine. Delaying thirst is one of the uses of the medicinal product of this plant which has been emphasized in Iranian traditional medicine though it was not proven scientifically. Accordingly, the present study aimed to investigate the effect of PO product on thirst.

**Materials and Methods:**

In this research, two main Set of experiments were considered: acute water deprivation group and chronic water restriction group. The urine parameters analyzed were osmolality, and sodium, and potassium concentration, and blood parameters evaluated included blood urea nitrogen, creatinine, osmolality, and sodium, and potassium concentration. The PO dosages were 50, 100 and 200 mg/kg.

**Results:**

The findings showed that the effects of PO 100 and 200 (mg/kg) on blood and urine parameters were greater than that of PO 50 mg/kg, but there were no significant differences between them.

**Conclusion:**

In general, these findings indicate that PO extract can play an important role in reducing thirst symptoms most likely by affecting intra- and extra-cellular environments. Also, it is recommended to study the beneficial effects of this plant on diseases that lead to hypokalemia or blood potassium depletion.

## Introduction

*Portulaca oleracea* L. (PO), as a valuable species, belongs to the Portulacaceae family. Furthermore, this plant, which is used as a vegetable, is grown in all parts of Iran and has wide acceptability as a common weed in farmlands (Akhoundzadeh, 1999 ▶; Radhakrishnan et al., 2001 ▶; Zhou et al., 2015). From the perspective of Traditional Iranian Medicine, the temperament of PO is cold and moist (Hamedi et al., 2019 ▶). 

In the west of Iran, all parts of PO are used as a stomach tonic and an anti-parasite remedy (Ghasemi Pirbalouti et al., 2013 ▶; Mosaddegh et al., 2012 ▶). 

In addition, PO has been described as an antimicrobial plant (Dan, 2006 ▶), a liver protector (Eidi et al., 2015 ▶), and an antioxidant (Karimi et al., 2011 ▶). Further, it is effective against the intestinal worm and has anti-inflammatory, muscle-relaxant (Parry et al., 1993), and contraceptive effects (Hanumantapa et al., 2014 ▶). Additionally, it prevents heart attack, enhances the immune system (Hozayen et al., 2011 ▶), acts as a blood purifier and thirst quencher (Amiri and Joharchi, 2013 ▶; al-Nafis, 1999 ▶), and has therapeutic application in rectal and mouth ulcers such as hemorrhoids and constipation (Simopoulos et al., 1998 ▶; Schumann, 2001 ▶; Kumar et al., 2008 ▶). In eastern Mallorca, it is used to regulate blood pressure (Cario and Wallis, 2012 ▶). 

In some European countries like Italy, Turkey, and Greece, PO has been used to treat a variety of diseases such as headache, stomach intestine and kidney pains, intestinal worms, dysentery, urogenital infections, urinary inflammations, scurvy, fever, and hemorrhoids (Bosi et al., 2009 ▶; Simopoulos, 2004 ▶; Brussell, 2004 ▶; Cakilcioglu and Turkoglu, 2010 ▶).

Further, the seeds and leaves of PO have long been used as a medicinal agent in the Central Asian and Middle Eastern countries. For example, PO is applied as an antidiarrheal agent and for the treatment of throat infection, liver and gastrointestinal problems, and inflammatory diseases in Afghanistan and Saudi Arabia (Al-Asmari, 2014 ▶). 

Kaveh et al. (2017) ▶ studied the effects of PO on bronchoalveolar lavage fluid, total protein levels, as well as phospholipase A2 and IgE and indicated its anti-inflammatory and immunomodulatory effects were equal to or greater than those of dexamethasone at studied concentrations.

Different studies indicated that the fruits and leaves of other plants like *Solanum nigrum* L., *Azadirachta indica *A. Juss*., Ficus bengalensis, Ficus religiosa, Murraya koenigii, Ziziphus jujube, Citrullus lanatus, Grewia tenax, Murraya koenigii, *and* Maytenus emarginata* are used for treating the thirst (Teklehaimanot et al., 2015 ▶, Kumar et al., 2008 ▶, Handral et al., 2012 ▶ and Rajbir Kaur, 2015).

Anti-thirst effects of PO was shown (Amiri and Joharchi, 2013 ▶). Several studies from different countries also reported the effect of PO on thirst, including Al-Nafis, (1999) ▶, Irawan et al. (2003) ▶, Keter and Mutiso, (2011) ▶, Handral et al. (2012) ▶, Syed et al. (2016) ▶, and Mastud et al. (2018) ▶ from South Africa, Indonesia, Kenya, India, and India, respectively.

The components of the PO seed are luteolin (Xu et al., 2006 ▶), myricetin (Xu et al., 2006 ▶), quercetin (Xu et al., 2006 ▶), genistein (Zhu et al., 2010 ▶), genistin (Zhu et al., 2010 ▶), dopamine (Yu et al., 2005 ▶), noradrenalin (Chen et al., 2003 ▶), oleraceins (Xiang et al., 2005 ▶), adenosine (Xiang et al., 2005 ▶), kaempferol (Xu et al., 2006 ▶), and apigenin (Xu et al., 2006 ▶), 

 Bekkevold et al. (2013) ▶ investigated various regimens of water deprivation and restriction and their effects on the appearance, attitude, and physiologic indicators of dehydration by acute water deprivation and chronic water restriction in static and ventilated cages.

Although various studies have indicated the use of PO, none of them have provided any scientific justification regarding its role in delaying the thirst. Thus, the present study investigated the PO extract, as well as various regimens of water deprivation and restriction and their effects on the selection of key physiologic indicators of dehydration in mice. Hopefully, this research is helpful in designing future clinical studies and developing new pharmaceuticals containing PO.

## Materials and Methods

**Animals**

Male mice of about the same weight and age (20-25 g and within the age range of 5-8 weeks) were obtained from Pasteur Institute of Iran. In addition, these animals were selected for this project because of their availability, size, low cost, ease of handling, and a powerful application for modeling human diseases (Justice et al., 2011 ▶). They were kept in a room with natural light at about 22^°^C (Bekkevold et al., 2013 ▶). Further, all mice had free access to water and chow for 1 week although access was prevented or restricted during the experiment time.

**The extraction process of** ***Portulaca oleracea*** **L.** **(PO)**

The seed of PO was purchased from a herbal market in Tehran and authenticated by Dr. Mozhgan Mehri and a voucher specimen 001/001/151 was deposited at the herbarium of Gorgan University of Agricultural Sciences and Natural Resources. The powdered seeds were then extracted using 70% ethanol by maceration for 7 days, followed by filtering and evaporating the extract under reduced pressure (Ahangarpour et al., 2018 ▶). It should be mentioned that 50, 100, and 200 mg/kg doses of PO were chosen for this experiment in order to find the better dose of PO (Ahangarpour et al., 2018 ▶; Baradaran Rahimi et al., 2005 ▶)

**Thirst induction protocol**

**Acute water deprivation**

This main group consisted of 4 subgroups. Mice used pure water (without PO), water mixed with 50 mg/kg of PO, water mixed with 100 mg/kg of PO, and water mixed with 200 mg/kg of PO in groups 1 (control group), 2, 3 and 4, respectively. Then, blood and urine samples were collected every 12 hours (0-, 12-, 24-, and 36-hr) according to the study of Bekkevold et al. (2012) ▶. 

**Chronic water restriction**

This group included 2 subgroups and each subgroup consisted of 4 subgroups. In these groups, mice had access to water but in different amounts encompassing 75% (75% water ration) and 50% (50% water ration) of daily water intake. All blood and urine samples were taken at 9 am for 7 days (Bekkevold et al., 2012 ▶). Then, the normal water intake for a 24-hr period was determined during the acclimation period by measuring the daily water intake of each mouse (approximately 6.8 ml).

1. Mice used 75% of the daily water intake: In group A (control group), mice used 75% water ration and those in group B used 75% water ration mixed with 50 mg/kg of PO. In addition, groups C and D used 75% water ration mixed with 100 mg/kg and 200 mg/kg of PO, respectively.
2. Mice used 50% of the daily water intake: Group A (control group) mice used 50% water ration and those in group B used 50% water ration mixed with 50 mg/kg of PO. Further, mice in groups C and D used 50% water ration mixed with 100 mg/kg and 200 mg/kg of PO, respectively.

**Quantification of total phenol**

The total phenolic contents of plant extracts were estimated by using the Folin-Ciocalteu assay (Karimian et al., 2013 ▶). 

**Figure 1:**
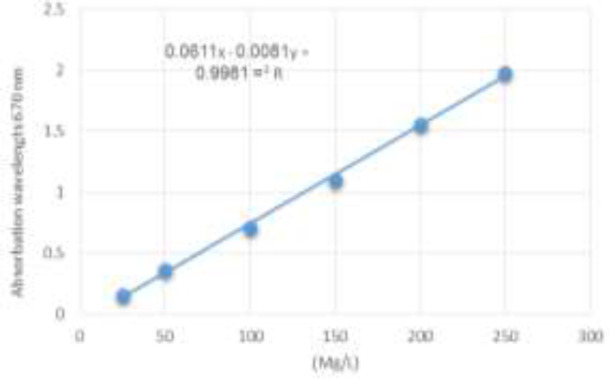
The standard curve of gallic acid equivalent

**Hematology and urinary analyses**

The blood sample was taken from the heart, and urine was collected from the bladder at the postmortem examination (Bekkevold et al., 2012 ▶), followed by measuring urinary and blood variables including potassium, sodium, blood urea nitrogen (BUN), creatinine, and osmolality (Bekkevold et al., 2012 ▶; Kavouras, 2002 ▶; Oppliger and Bartok, 2002; Shirreffs, 2003). Moreover, plasma and urine osmolality was measured by using a vapor pressure micro osmometer. More precisely, both plasma and urine sodium and potassium concentrations, as well as plasma BUN and creatinine were estimated by a flame photometer (IL 943 flame photometer) and an automated chemistry analyzer (VetAce, ALFAWasserman), respectively (Bekkevold et al., 2012 ▶).

**Statistical analysis**

The Anderson Darling normality test was done before data analysis (Jafari Foutami and Shaidai Karkaj, 2019). Eventually, the data were analyzed by using one-way ANOVA with *post-hoc* contrasts according to the Newman-Kuels test by SPSS 19 (Bekkevold et al., 2012 ▶). 

## Results

**Acute water deprivation**

All mice had similar body weights at the start of the deprivation period (21-24 g). Osmolality, and sodium, and potassium concentration increased by deprivation and was significantly (p<0.05) higher than that in mice that used 200 and 100 mg/kg of *P. oleracea *after 48 hr. The corresponding values of urinary osmolality and sodium and potassium concentrations are shown in Tables 1, 2, 3, respectively. The obtained data demonstrated that mice that used 200 mg/kg of PO, had the lowest sodium while the control mice had the highest sodium concentration (Figure 3). 

Figure 4 shows potassium concentration differences among 4 study groups. Animals that used PO had the lowest potassium although there were no significant differences between them and the control group after 12 hr deprivation. 

**Table 1:** Comparison of Osmolality (mOsm/kg) between different amounts of PO in acute water deprivation

Groups Duration (h)	Control	PO 50 (mg/kg)	PO 100 (mg/kg)	PO 200 (mg/kg)
0	1360.33±2.45	1353.33±3.69	1343.33±4.90	1346.33±3.34
12	1400±6.17	1367.67±9.91^$ &^	1361.66±7.88^# $^	1349±3.84^# &^
24	1419.66±4.90	1400±3.74^&^	1394.33±.45^#^	1376±6.17
36	1435.33±3.15^#^	1416.33±4.14^* &^	1408±5.56^# $^	1400±3.28^&^

* Within row, * shows control rats; # shows PO 50 (mg/kg); & shows PO 100 (mg/kg) and $ shows PO 200 (mg/kg). Symbols show the differences between groups in desired times. Data are expressed as mean±SD.

**Table 2:** Comparison of sodium concentration (mEq/L) between different amounts of PO in acute water deprivation

Groups Duration (h)	Control	PO 50 (mg/kg)	PO 100 (mg/kg)	PO 200 (mg/kg)
0	187.67±3.07	169.67±7.78	164.66±6.4	170.33±5.6
12	318±1.73	301.67±5.65^&^	294.66±4.7^#^	274.33±5.8
24	352.33±4.65^#^	341.67±3.32^* &^	327.66±5.23^& $^	317±4.04^&^
36	421.33±10.5^#^	378±6.09^* &^	384.33±8.6^* #^	360±27.5

* Within row, * shows control rats; # shows PO 50 (mg/kg); & shows PO 100 (mg/kg) and $ shows PO 200 (mg/kg). Symbols show the differences between groups in desired times. Data are expressed as mean±SD.

**Table 3:** Comparison of potassium concentration (mEq/L) between different amounts of PO in acute water deprivation

Groups Duration (h)	Control	PO 50 (mg/kg)	PO 100 (mg/kg)	PO 200 (mg/kg)
0	7.66±0.43	5.66±0.27^&^	5.67±0.37^#^	3.67±0.39
12	8.33±0.24	7.33±0.44	7.67±0.31	7.67±0.21
24	10.33±0.22	9±0.18	7.67±0.33^$^	7.67±0.34^&^
36	11±0.07	9.33±0.32^& ^$	8.33±0.3^# ^$	8.33±0.6^# ^&

* Within row, * shows control rats; # shows PO 50 (mg/kg); & shows PO 100 (mg/kg) and $ shows PO 200 (mg/kg). Symbols show the differences between groups in desired times. Data are expressed as mean±SD.

As depicted in Figure 5, mice that used PO had the lowest amount of blood urea nitrogen (BUN) although the control group had the highest BUN. 

Based on Figure 6, the control group had the highest creatinine and mice that used 200 (mg/kg) of the PO had the lowest amount of creatinine.

**Chronic water restriction**

**(**50% of the normal daily intake**) **Tables 4, 5 and 6 provide data regarding the urinary variables that were measured in chronic water restriction (50%).

**Figure 2:**
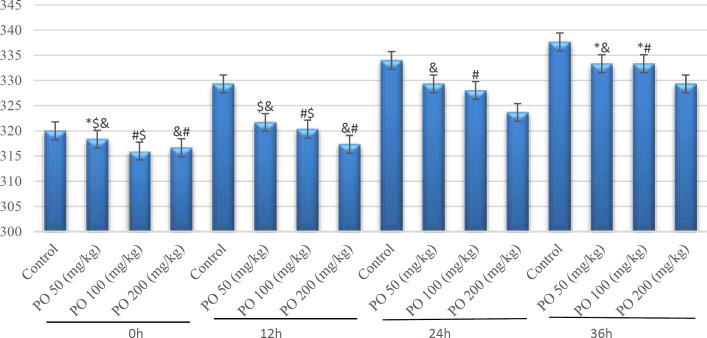
Comparison of plasma osmolality (mOsm/kg) between different amounts of PO in acute water deprivation. * shows control rats; # shows PO 50 (mg/kg); & shows PO 100 (mg/kg) and $ shows PO 200 (mg/kg). Symbols show the differences between groups in desired times

**Figure 3:**
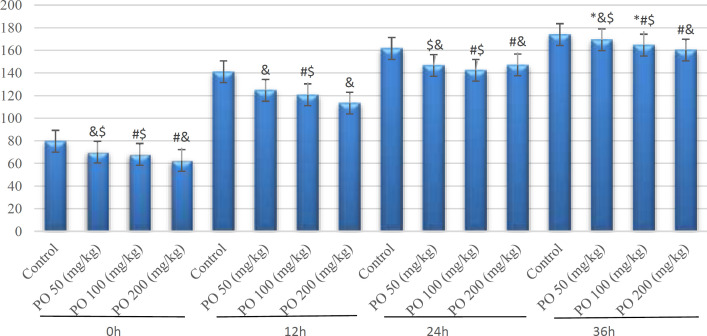
Comparison of plasma sodium concentration (mEq/L) between different amounts of **PO** in acute water deprivation. * shows control rats; # shows PO 50 (mg/kg); & shows PO 100 (mg/kg) and $ shows PO 200 (mg/kg). Symbols show the differences between groups in desired times

**Figure 4:**
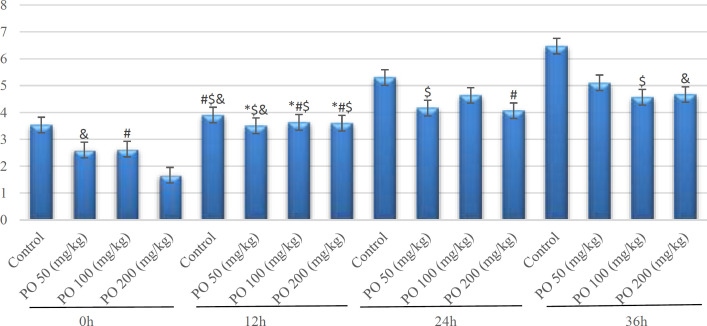
Comparison of plasma potassium concentration (mEq/L) between different amounts of PO in acute Water deprivation. * shows control rats; # shows PO 50 (mg/kg); & shows PO 100 (mg/kg) and $ shows PO 200 (mg/kg). Symbols show the differences between groups in desired times

**Figure 5:**
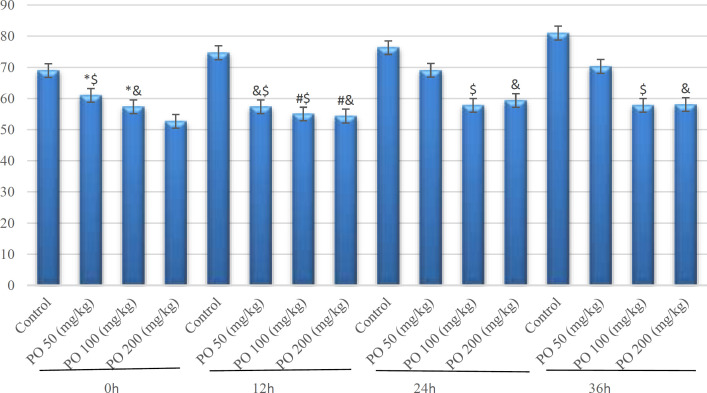
Comparison of BUN (mg/dL) between different amounts of PO in acute water deprivation. * shows control rats; # shows PO 50 (mg/kg); & shows PO 100 (mg/kg) and $ shows PO 200 (mg/kg). Symbols show the differences between groups in desired times

**Figure 6:**
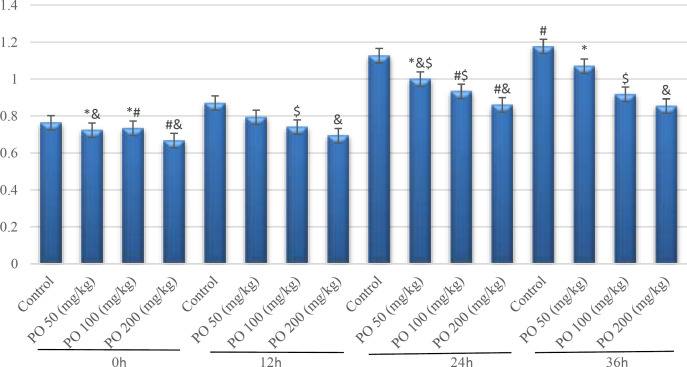
Comparison of plasma creatinine (mg/dL) between different amounts of PO in acute water deprivation. * shows control rats; # shows PO 50 (mg/kg); & shows PO 100 (mg/kg) and $ shows PO 200 (mg/kg). Symbols show the differences between groups in desired times

**Table 4:** Comparison of Osmolality (mOsm/kg) between different amounts of PO in chronic water restriction (50%).

Day							
	First	Second	Third	Fourth	Fifth	Sixth	Seventh	
Control	436±1.59	442±3.44	447±5.20	448.66±1.52^#$&^	451.33±6.17^#$&^	453.66±3.61^#^	458.33±12.4^#^	
PO 50(mg/kg)	435±3.18	437.66±4.49	449±10.78	451±3.61^&*^	463.66±11.10^*^	464.33±1.16^*^	473±8.84^*^	
PO 100 (mg/kg)	434±2.68	433.66±5.42	435±4.47	431.66±0.76^*$#^	430.33±7.98^*$^	419.33±5.08^$^	419.33±2.89$	
PO 200(mg/kg)	433±3.92	431.66±1.52	433.66±2.6	429.66±10.98^#&^	427.33±4.77^*&^	422±6.67^&^	418.33±1.52^&^	

*Within the column, * shows control rats; # shows PO 50 (mg/kg); & shows PO 100 (mg/kg) and $ shows PO 200 (mg/kg). Symbols show the differences between groups in desired days. The values represent the mean±SD.

**Table 5:** Comparison of sodium concentration (mEq/L) between different amounts of PO in chronic water restriction (50%).

				**Day**			
Groups	First	Second	Third	Fourth	Fifth	Sixth	Seventh
Control	324.67±2.78^#^	335±3.85^#^	333.33±2.67^#^	341±2.04^#^	336.66±0.77^#^	344.33±4.08^#^	352.33±2.78^#^
PO 50 (mg/kg)	314.67±2.67^*$&^	328.66±1.33^*^	332±5.05^*^	333.33±2.6^*^	340±7.07^*^	345±8.12^*^	350.33±6.25^*^
PO 100(mg/kg)	310±3.53^# $^	314.66±1.3	316.33±3.36^$^	313.33±5.4^$^	312.66±4.81^$^	310.33±5.82^$^	307.66±4.6^$^
PO 200(mg/kg)	307.67±4^# &^	307±2.04	314.66±2.67^&^	317.66±4.29^&^	319.33±5.34^&^	316.66±6.72^&^	317±7.4^&^

*Within the column, * shows control rats; # shows PO 50 (mg/kg); & shows PO 100 (mg/kg) and $ shows PO 200 (mg/kg). Symbols show the differences between groups in desired days. The values represent the mean±SD.

**Table 6:** Comparison of potassium concentration (mEq/L) between different amounts of PO in chronic water restriction (50%).

Groups	First	Second	Third	Fourth	Fifth	Sixth	Seventh
Control	14.66±0.27	14.33±0.42^#&^	14.33±0.72	16±0.10^#&^	17.66±0.37^#^	18.33±0.58^#&^	19±0.55^#^
PO 50 (mg/kg)	12±0.47^& $^	13.33±0.25^*$&^	13±0.63	14±0.65^*$&^	14.66±0.75^*$&^	15±1.47^*$&^	15.66±0.68^*&^
PO 100(mg/kg)	12.66±0.18^$#^	12.66±0.65^*#$^	13±0.68	14.33±1.10^*#$^	13.33±1.63^$#^	15.66±1.38^*$#^	15±0.9^#^
PO 200(mg/kg)	11.33±0.81^$#^	12±0.68^$#^	12.66±0.63	12±0.45^&$^	12±0.36^#&^	11.33±1.31^$#^	11.33±1.18

*Within the column, * shows control rats; # shows PO 50 (mg/kg); & shows PO 100 (mg/kg) and $ shows PO 200 (mg/kg). Symbols show the differences between groups in desired days. The values represent the mean±SD.

As shown, the lowest concentration of sodium (307.66 mEq/L) was found on the seventh day for PO 100 mg/kg whereas the highest sodium concentration (352.33 mEq/L) was related to the control group on the seventh day. The lowest concentration of potassium (11.33 mEq/L) was found on the first, sixth, and seventh day for PO 200 mg/kg. On the other hand, the control group had the highest potassium concentration (19 mEq/L) on the seventh day. The lowest concentration of urine osmolality (418.33 mOsm/kg) was observed on the seventh day for PO 200 mg/kg while the control group had the highest osmolality (458.33 mOsm/kg) on day seven. Similarly, the control group and the group that used 50 mg/kg of the PO demonstrated no significant differences in three first day. 

**Figure 7:**
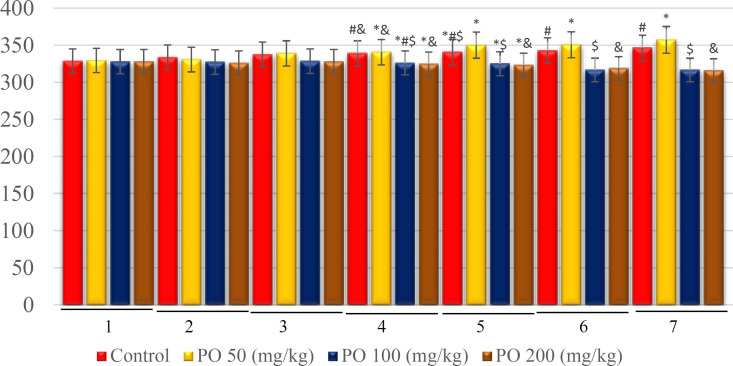
Comparison of plasma osmolality (mOsm/kg) between different amounts of PO in chronic water restriction (50%). * shows control rats; # shows PO 50 (mg/kg); & shows PO 100 (mg/kg) and $ shows PO 200 (mg/kg). Symbols show the differences between groups in desired days

**Figure 8:**
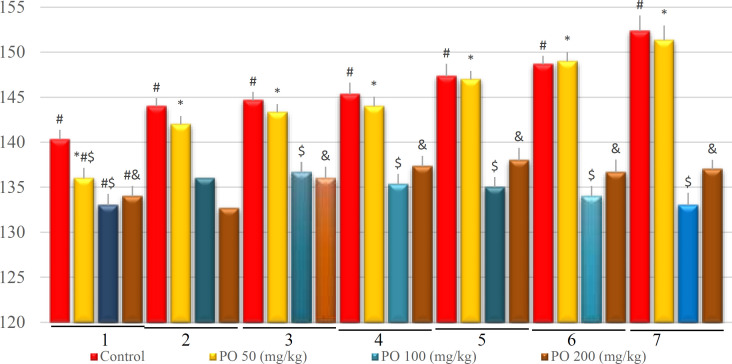
Comparison of plasma sodium concentration (mEq/L) between different amounts of PO in chronic water restriction (50%). * shows control rats; # shows PO 50 (mg/kg); & shows PO 100 (mg/kg) and $ shows PO 200 (mg/kg). Symbols show the differences between groups in desired days

**Figure 9:**
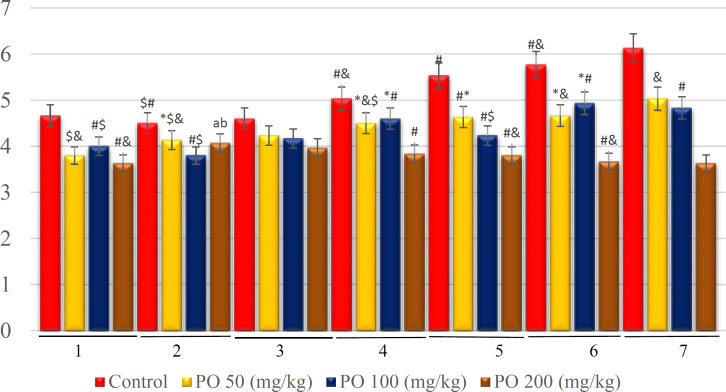
Comparison of plasma potassium concentration (mEq/L) between different amounts of PO in chronic water restriction (50%). * denotes control rats; # denotes PO 50 (mg/kg); & denotes PO 100 (mg/kg) and $ denotes PO 200 (mg/kg). Symbols show the differences between groups in desired days

**Figure 10:**
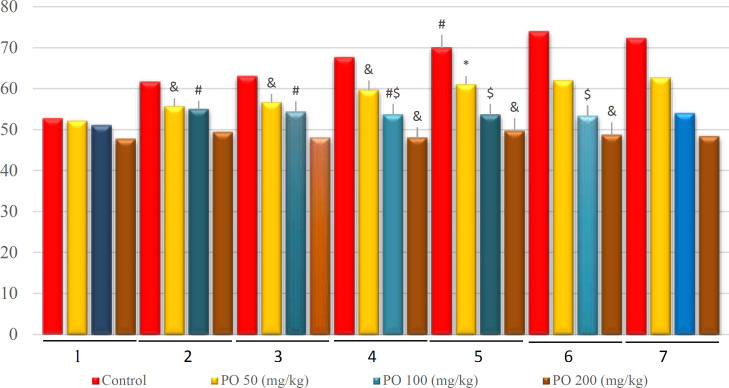
Comparison of BUN (mEq/L) between different amounts of PO in chronic water restriction (50%). * denotes control rats; # denotes PO 50 (mg/kg); & denotes PO 100 (mg/kg) and $ denotes PO 200 (mg/kg). Symbols show the differences between groups in desired days

**Figure 11:**
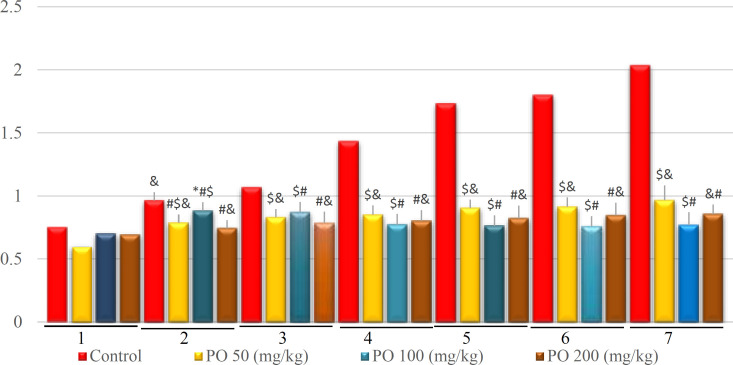
Comparison of plasma creatinine (mg/dL) between different amounts of PO in Chronic Water Restriction (50%). * denotes control rats; # denotes PO 50 (mg/kg); & denotes PO 100 (mg/kg) and $ denotes PO 200 (mg/kg). Symbols show the differences between groups in desired days

Figures 7-11 depict the plasma variables measured based on chronic water restriction (50%). There were no significant differences among the groups during the first three days. Although the plasma osmolality in the groups that took PO were less than that of the other groups in study days (Figure 7). 

The findings indicated that no significant differences were detected during seven days between the control group and the mice that consumed 50 mg/kg of PO and their sodium concentrations were extremely more than two other groups (Figure 8). 

Based on the statistical analysis, plasma potassium concentration in group used 200 (mg/kg) was lower than the other three groups but there were no significant differences between this group and the group that used 100 mg/kg of PO except on day seven. The control group had the highest concentration of potassium (Figure 9). 

The BUN changes are shown in Figure 10. BUN was not significantly affected by the amount of PO on the first day, but on other days, there were significant differences between different groups, and BUN had the lowest amount in mice that used 200 mg/kg of PO. 

Contrarily, no significant differences were found between the group that consumed PO during the experimental period, and plasma creatinine was lower in the group that used 100 mg/kg of PO in the last four days (Figure 11). 

**Chronic water restriction**

**(**75% of the normal daily intake**)**

Table 7, 8 and 9 present the urinary variables that were measured in chronic water restriction (75% ration). As shown, the lowest concentration of sodium (98 mEq/L) was observed on the seventh day for 200 mg/kg and the control group had the highest sodium concentration (130 mEq/L) on day seven (Table 7). The lowest concentration of potassium (3.03 mEq/L) was related to the second and third day for 200 mg/kg and the control group had the highest potassium concentration (10.5 mEq/L) on the second day (Table 8). The lowest concentration of urine osmolality (368 mOsm/kg) was found on the first day for PO 200 (mg/kg) and the control group had the highest osmolality concentration (567.33 mOsm/kg) on the seventh day (Table 9). 

**Table 7:** Comparison of Osmolality (mOsm/kg) between different amounts of PO in chronic water restriction (75%)

Day							
Groups	First	Second	Third	Fourth	Fifth	Sixth	Seventh	
Control	558±6.02	560±28.79^#^	563±9.84	563.33±5.69	563.66±3.48	564.66±10.49	567.33±14.8	
PO 50 (mg/kg)	495±13.8	517.33±9.38*	518.33±12.41	521±5.85	522.66±3.75	523.33±10.1	523.33±7.05	
PO 100 (mg/kg)	395.33±16.8^$^	399±5.13^$^	407.33±3.84^$^	405±2.64^$^	404.66±5.69^$^	404.66±2.96^$^	405.33±4.17^$^	
PO 200 (mg/kg)	386±13.2^&^	390.33±22.73^$^	396.66±2.40^&^	400.66±5.20^&^	405.66±10.36^&^	398.66±14.8^&^	412±10.21^&^	

*Within the column, * shows control rats; # shows PO 50 (mg/kg); & shows PO 100 (mg/kg) and $ shows PO 200 (mg/kg). Symbols show the differences between groups in desired days. The values represent the mean±SD.

**Table 8:** Comparison of sodium concentration (mEq/L) between different amounts of PO in chronic water restriction (75%).

				**Day**			
Groups	First	Second	Third	Fourth	Fifth	Sixth	Seventh
Control	126.33±1.45^#^	126±1.15^#^	126.66±1.85^#^	127.66±1.85^#^	128.33±1.2^#^	129±2.08^#^	130±2.64^#^
PO 50 (mg/kg)	123.33±1.42^&*^	123±1.52^*^	123.66±2.33^*^	123±1.15^*^	123.33±1.21^*^	123.66±0.66*	124±4.04^*^
PO 100(mg/kg)	116.6±6.1^#$^	111.66±0.8	109±6.08^$^	108.33±1.20^$^	112.33±5.20	108±5.50^$^	106±4.61^$^
PO 200(mg/kg)	107±4.71^&^	104.33±3.28	101.66±2.40^&^	101.33±10.3^&^	99.66±3.32	100.66±1.20^&^	98±3.6^&^

*Within the column, * shows control rats; # shows PO 50 (mg/kg); & shows PO 100 (mg/kg) and $ shows PO 200 (mg/kg). Symbols show the differences between groups in desired days. The values represent the mean±SD.

**Table 9:** Comparison of potassium concentration (mEq/L) between different amounts of PO in chronic water restriction (75%).

				**Day**			
Groups	First	Second	Third	Fourth	Fifth	Sixth	Seventh
Control	9.56±1.54^#^	10.5±0.74^#^	10.3±2.21^#^	10.43±1.37^#^	9.86±1.21^#^	9.5±0.28^#^	9.1±0.49^#^
PO 50 (mg/kg)	9.3±0.7^*^	9.63±1.28^*^	9.7±0.85^*^	9.43±1.66^*^	9.96±2.54^*^	9.63±2.05*	8.96±0.60^*^
PO 100(mg/kg)	4.13±0.31^$^	4.93±0.71^$^	4.63±0.40^$^	4.63±0.56^$^	4.03±0.59^$^	3.9±0.68^$^	4.46±0.80^$^
PO 200(mg/kg)	3.66±0.29^&^	3.03±0.29^&^	3.03±0.18^&^	3.36±2.92^&^	3.93±0.43^&^	4±0.1^&^	3.46±0.23^&^

*Within the column, * shows control rats; # shows PO 50 (mg/kg); & shows PO 100 (mg/kg) and $ shows PO 200 (mg/kg). Symbols show the differences between groups in desired days. The values represent the mean±SD.

**Figure 12:**
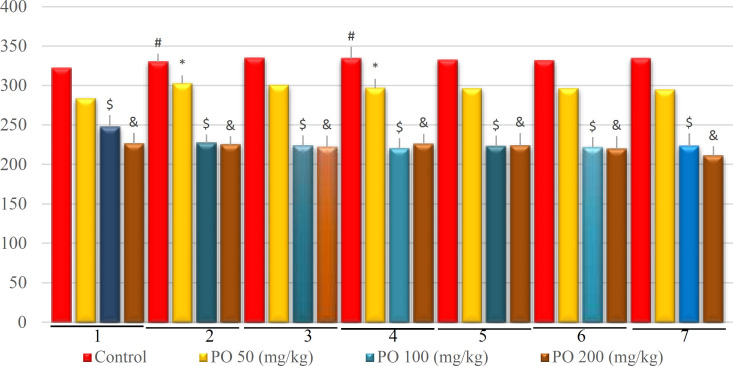
Comparison of plasma osmolality (mOsm/kg) between different amounts of PO in chronic water restriction (75%). * denotes control rats; # denotes PO 50 (mg/kg); & denotes PO 100 (mg/kg) and $ denotes PO 200 (mg/kg). Symbols show the differences between groups in desired days

**Figure 13:**
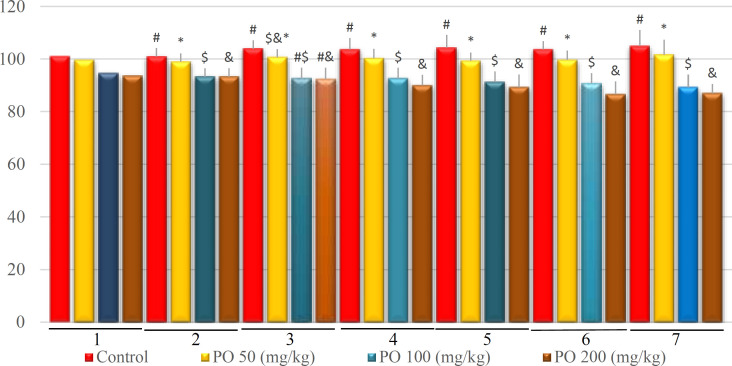
Comparison of plasma sodium concentration (mEq/L) between different amount of PO in chronic water restriction (75%). * denotes control rats; # denotes PO 50 (mg/kg); & denotes PO 100 (mg/kg) and $ denotes PO 200 (mg/kg). Symbols show the differences between groups in desired days

**Figure 14:**
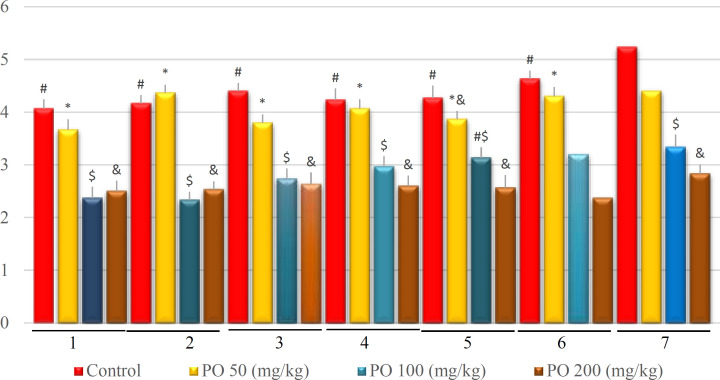
Comparison of plasma potassium concentration (mEq/L) between different amount of PO in chronic water restriction (75%). * denotes control rats; # denotes PO 50 (mg/kg); & denotes PO 100 (mg/kg) and $ denotes PO 200 (mg/kg). Symbols show the differences between groups in desired days

**Figure 15:**
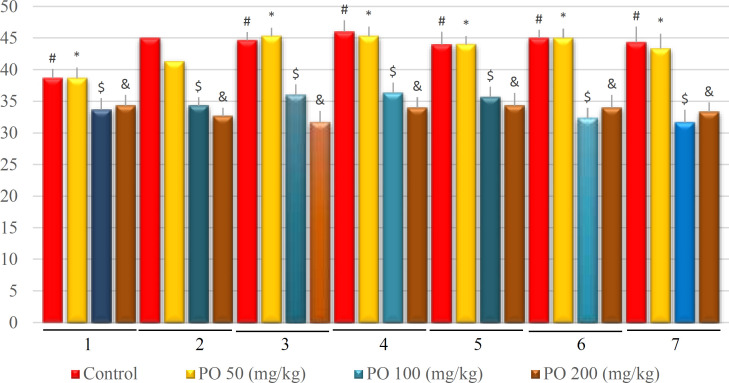
Comparison of BUN (mEq/L) between different amounts of PO in chronic water restriction (75%). ^*^ denotes control rats. * denotes control rats; # denotes PO 50 (mg/kg); & denotes PO 100 (mg/kg) and $ denotes PO 200 (mg/kg). Symbols show the differences between groups in desired days

**Figure 16:**
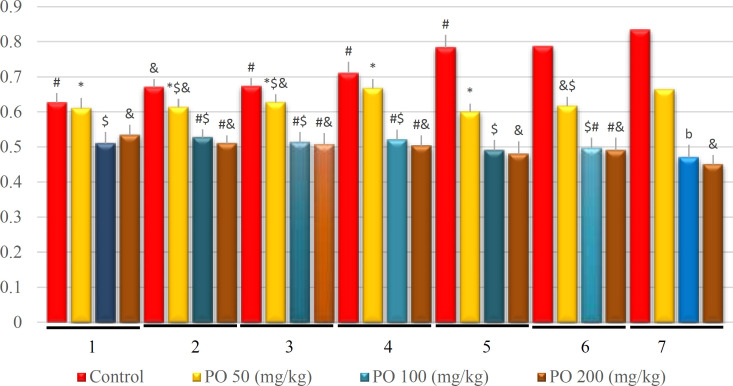
Comparison of plasma creatinine (mg/dL) between different amounts of PO in chronic water restriction (75%).* denotes control rats; # denotes PO 50 (mg/kg); & denotes PO 100 (mg/kg) and $ denotes PO 200 (mg/kg). Symbols show the differences between groups in desired days

Figures 12-16 illustrate the plasma variables measured based on chronic water restriction (75% ration) in this study. The plasma osmolality in the groups that used PO were less than that of the control on study days and the control group had a higher osmolality compared to the other groups (Figure 12). 

Similarly, there were no significant differences between the groups that used 100 and 200 mg/kg of PO except on the first day. In addition, no significant differences were found between the control group and those mice that used 50 mg/kg of PO, and their sodium concentration was extremely higher compared to the groups that used 100 and 200 (mg/kg) of PO (Figure13). Further, no significant differences were observed between the groups that consumed 100 and 200 mg/kg of the PO, and their potassium concentration was extensively higher than that of the other two groups (Figure 14).

 Furthermore, no significant differences of BUN were detected between the study groups on the first day, as well as between the groups that used 100 and 200 mg/kg of PO in the other six days (Figure 15). The findings further revealed that the group that used 100 and 200 (mg/kg) of the PO had lower creatinine and they did not have a significant difference compare to two other groups. The control group had the highest amount of creatinine (Figure 16).

## Discussion

Assessment of the effect of *Portulaca oleracea *L. extract on water deprivation requires an understanding of physiologic mechanisms that occur by inadequate water intake.

One of the most important driving factors of thirst is the increase of extracellular fluid osmolarity (osmotic thirst, hypernatremia) which causes intracellular dehydration in the center of thirst and, therefore, stimulates thirst (Amirian et al., 2016 ▶). Additionally, the second driving factors of thirst is decreasing the volume of extracellular fluid, and the independent reduction of arterial pressure also causes thirst, namely, hypovolemic thirst (Amirian et al., 2016). 

Thirst is influenced by changes in plasma osmolality and plasma sodium (Kavouras, 2002 ▶; Toth and Gardiner, 2000 ▶), as well as intracellular dehydration (Greenleaf, 1992 ▶). According to some studies, the perception of thirst increases when plasma osmolality and plasma sodium demonstrate an increase (Fitzsimons, 1976 ▶; Toth and Gardiner, 2000 ▶). 

After the deprivation of our mice, both plasma osmolality and sodium of the control group increased significantly as compared with PO groups, which is most likely because of the effect of PO on intracellular and extracellular water circulation. 

These changes in intracellular and extracellular environments demonstrate potential physiologic distress (Bekkevold et al., 2013 ▶). Further, changes in the intracellular environment can affect the activity of other hormones (Hohenegger et al., 1986). One of these hormones is aldosterone, which causes a decrease in the sodium output from the proximal tubules, preserving plasma sodium and driving the maintenance of body fluid by cellular dehydration (Rowland et al., 2004 ▶). The results of this study demonstrated that the sodium output was less in PO groups compared to the control group.

The oleraceins in PO have anti-inflammatory effects on lipopolysaccharide-stimulated macrophages. This component remarkably inhibited nitric oxide production and could dose-dependently decrease the secretions of interleukin 6, tumor necrosis factor α, nitric oxide, and prostaglandin E2 in cell culture supernatants, as well as the mRNA of cyclooxygenase-2 and inducible nitric oxide synthase (Li et al., 2016 ▶).

On the other hand, the reduced fluid intake that occurs in aging is due to increased COX-PGE_2_-mediated inflammation (Begg et al., 2020 ▶). Therefore, it seems that PO can prevent the increase of blood osmolality by this mechanism. In addition, the urine osmolality increases by decreasing body fluid (Armstrong et al., 2014 ▶) but the results of this study showed that PO could prevent an increase in urine osmolality. 

Other mechanisms of PO effects on thirst symptoms were mentioned by Oyedeji and Blariwa (2012) ▶, *P. oleracea* L. causes to increase the blood protein and therefore, it increases the capacity of the blood buffer and the body's fluids balance. 

Blood urea nitrogen (BUN) and creatinine increase when body fluid decreases in a normal situation (Blantz, 1998 ▶) while PO prevents the increase of BUN and creatinine and thus helps the body to resists against dehydration.

Based on the findings of the present study, PO extract has a thirst-quenching effect in different doses although PO 100 (mg/kg) seems to be more appropriate based on its cost. 

Accordingly, future studies must investigate pharmacological activities related to the traditional uses of PO, especially regarding its effect on body resistance to thirst. Moreover, clinical examinations should be run to evaluate the efficacy, safety, and suitable dosage of PO in order to develop safe and efficient dosage forms of this plant. Finally, it is recommended to study the beneficial effects of this plant on diseases that lead to hypokalemia or blood potassium depletion. Although our findings may not be directly applicable to all kinds of species, they represent a basic framework for using PO and other species to examine the thirst-quenching condition. 
